# Saccade Velocity Driven Oscillatory Network Model of Grid Cells

**DOI:** 10.3389/fncom.2018.00107

**Published:** 2019-01-10

**Authors:** Ankur Chauhan, Karthik Soman, V. Srinivasa Chakravarthy

**Affiliations:** Department of Biotechnology, Indian Institute of Technology Madras, Chennai, India

**Keywords:** saccades, grid cells, salience map, hippocampus, principal component analysis-PCA, oscillator

## Abstract

Grid cells and place cells are believed to be cellular substrates for the spatial navigation functions of hippocampus as experimental animals physically navigated in 2D and 3D spaces. However, a recent saccade study on head fixated monkey has also reported grid-like representations on saccadic trajectory while the animal scanned the images on a computer screen. We present two computational models that explain the formation of grid patterns on saccadic trajectory formed on the novel Images. The first model named Saccade Velocity Driven Oscillatory Network -Direct PCA (SVDON—DPCA) explains how grid patterns can be generated on saccadic space using Principal Component Analysis (PCA) like learning rule. The model adopts a hierarchical architecture. We extend this to a network model viz. Saccade Velocity Driven Oscillatory Network—Network PCA (SVDON-NPCA) where the direct PCA stage is replaced by a neural network that can implement PCA using a neurally plausible algorithm. This gives the leverage to study the formation of grid cells at a network level. Saccade trajectory for both models is generated based on an attention model which attends to the salient location by computing the saliency maps of the images. Both models capture the spatial characteristics of grid cells such as grid scale variation on the dorso-ventral axis of Medial Entorhinal cortex. Adding one more layer of LAHN over the SVDON-NPCA model predicts the Place cells in saccadic space, which are yet to be discovered experimentally. To the best of our knowledge, this is the first attempt to model grid cells and place cells from saccade trajectory.

## Introduction

A map that aids (Andersen et al., 2009) spatial navigation of an animal was believed to be represented in the hippocampal-entorhinal complex (O'Keefe and Dostrovsky, 1971; Taube et al., 1990a,b; Rolls, 1999; Solstad et al., 2008). Grid cells reported in the dorso-caudal medial entorhinal cortex (MEC), fire periodically such that the firing fields of the neuron form a hexagonal grid-like structure in the physical space in which the animal navigates. There is a general consensus that grid cells code for the distance of movement and hence they have been assigned the function of path integration which is essential for spatial navigation (Hafting et al., 2005). There are other spatial cells, fewer in number, like the place cells, border cells, view cells, speed cells etc., that code for one or other aspect of the ambient space (O'Keefe and Dostrovsky, 1971; Taube et al., 1990a,b; Rolls, 1999; Franzius et al., 2007; Solstad et al., 2008; Kropff et al., 2015). The aforementioned neurons are thought to collectively form an internal map of the external space in which the animal navigates.

Killian et al. (2012) reported hexagonal grid-like representations in the MEC of monkeys during mere visual exploration of a scene, even when the animal was not performing active navigation in the external space. Recordings were taken from neurons in Entorhinal Cortex (EC) and hippocampus of three head fixed monkeys, performing a free-viewing visual recognition task, the visual preferential looking task (VPLT; Jutras et al., 2009; Jutras and Buffalo, 2010). Monkeys were shown a sequence of novel images on a computer screen. The displayed images consisted of diverse themes like art, animals, landscape, and people. These static images were scanned by the monkey using a dynamic sequence of fixation. Neurons in MEC emitted action potentials on the multiple fixation points, as the monkey scanned the images; the firing field resembled the canonical grid cells in navigation with distinct hexagonal firing fields (Hafting et al., 2005; Killian et al., 2012). The grid representations generated by the saccadic movements resembled those of spatial navigation in many respects. Gridness scores of saccade grids were comparable with those of the navigation grids. Saccade grids also exhibited theta modulation in its activity. The gradient of the grid scale along the dorso-ventral axis of MEC was reported in the case of saccade grid too and Local Field Potentials (LFPs) showed theta band oscillations (Killian et al., 2012).

Apart from the grid representations on saccade trajectory, experimental studies reported neurons coding for the direction of saccade movement viz. saccade direction (SD) cells (Killian et al., 2015). These cells are analogous to head direction cells, corresponding to spatial navigation, reported in the rat postsubicular region (Taube et al., 1990a,b). SD cells were reported from the posterior EC of two monkeys performing a visual recognition memory task (Manns et al., 2000; Jutras and Buffalo, 2010). During the tasks, the monkeys were allowed to freely scan the complex visual images. These neurons were reported to be preferentially active when the eye movement was made in a particular direction. SD cells showed a gradient in their tuning width such that with the increase in distance from rhinal sulcus, the width of tuning of individual neuron to preferred saccade direction also increased.

There exists a large corpus of literature on the computational models of the grid representation during active navigation. Models of grid cells generally fall into two categories: oscillatory interface models (OI) and attractor network models. Proposed by O'Keefe and Recce (1993), spatial periodicity in OI models arises as a result of the interference between velocity-controlled dendritic and constant somatic oscillations (Burgess et al., 2007) or from purely velocity-driven oscillators (Zilli and Hasselmo, 2010; Burgess and O'Keefe, 2011). In the case of neural attractor model, spatial periodicity arises due to the intrinsic symmetry of the attractor network (Fuhs and Touretzky, 2006; Burak and Fiete, 2009). A hybrid approach has also been used wherein these two methods were combined to explain spatial periodicity (Bush and Burgess, 2014). However, the aforementioned models are based on a biologically unrealistic assumption such as 60° phase difference in the head direction inputs of the oscillatory interference model, or the assumption of the weight connectivity of the attractor network having special symmetry conditions (Mhatre et al., 2012).

The proposed model for the neural representations on the saccade trajectory is built on the principles derived from a recent model that used multisensory modalities to explain the formation of spatial representations during active navigation (Soman et al., 2018a). It was a hybrid neural model that used both oscillatory and rate coded dynamics. The model captured the empirically reported spatial cell representations and the influence of multiple sensory modalities on such representations. We take the general principle of this model and currently adapt it to explain the grid cell representations in saccade trajectory.

We present Saccade Velocity Driven Oscillatory Network (SVDON) model that captures the empirically reported neural representations on saccade trajectories and also makes novel predictions on saccade representation. The input image presented to the SVDON is passed through four stages viz: saccade generation, saccade direction encoding, path integration, and unsupervised neural network stage which are explained in detail in the methods section.

## Methods

**Figure 1:**
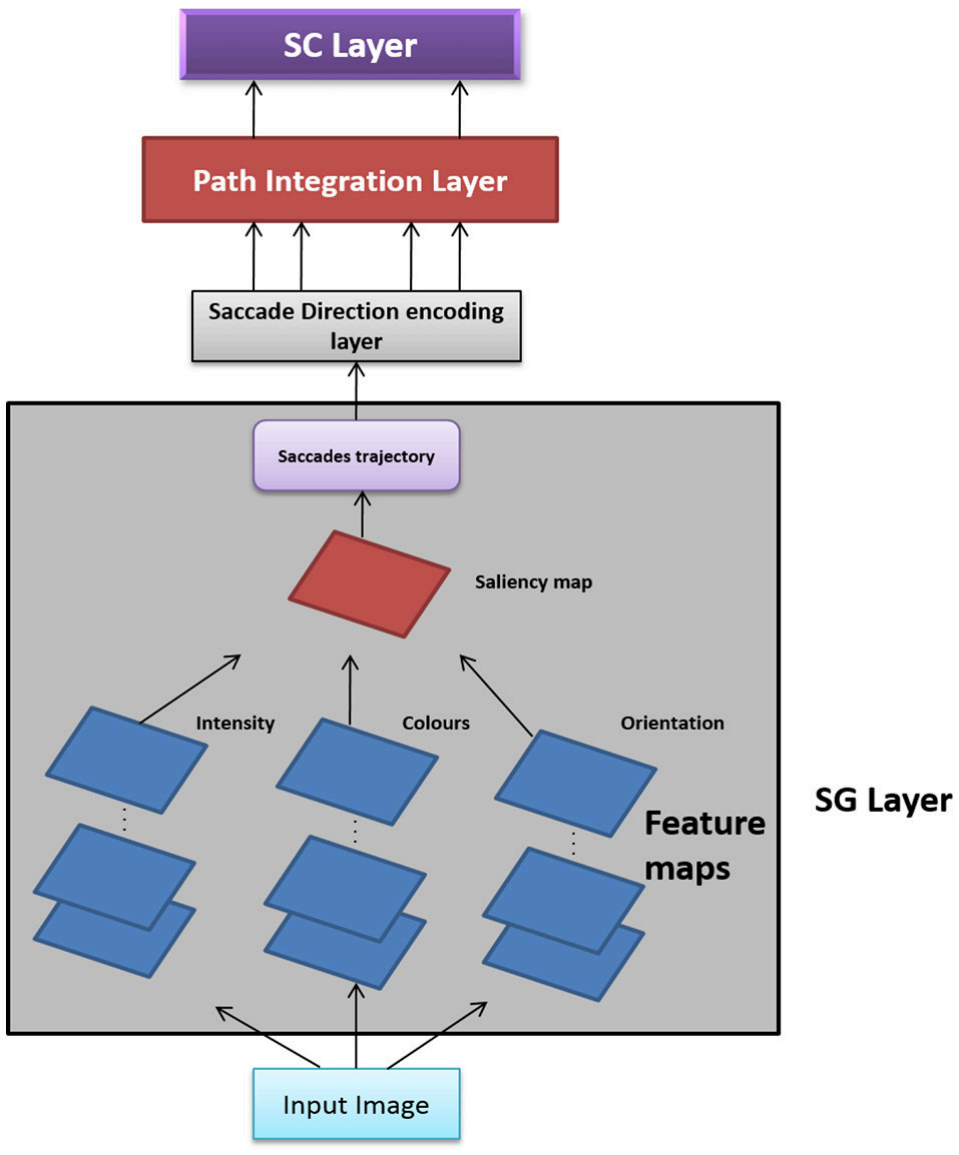
Model Architecture of SVDON: The model consists of a saccade generation stage, Saccade Direction Encoding stage, Path Integration stage and the output SC layer. (No copyright permission is required).

In this Section, we present two versions of Saccade Velocity Driven Oscillatory Network (SVDON) model (Shown in Figure 1): SVDON Direct PCA (SVDON-DPCA) and SVDON-Network PCA (SVDON-NPCA). Both the models capture the responses of grid cells to saccadic trajectories. SVDON-DPCA model consists of a Saccade Generating stage (SG), Saccade Direction encoding layer (SD), Path Integration layer (PI), and Spatial Cell layer (SC). SVDON-NPCA model shares a similar architecture except SD layer and SC layer, where it uses the self-organizing map (SOM) and Lateral Anti-Hebbian Network -Spatial cell layer (LAHN-SC) as the output layer. SVDON-NPCA is a network extension of SVDON-DPCA.

### SVDON-DPCA Architecture

The information flow in the model can be described as follows. The images to be scanned are given as input to the SG stage of the SVDON model to produce saccade trajectory. Velocity vectors are computed from the generated saccade trajectory. These velocity vectors are further passed on to the SD layer, where each neuron encodes for saccadic direction. Responses from the SD layer are passed on to the PI layer via one-to-one connection. Each neuron in the PI layer is a phase oscillator that receives SD response as its input. This further encodes the saccade position information along that direction component as the phase of the respective oscillator. The PI layer projects to the output SC layer which exhibits grid-like pattern by extracting the principal components of the oscillatory response. Each stage of the model is described below.

#### Saccade Generation (SG) Stage

The model used for saccade generation is a bottom-up model of attention that is based on locating the single most salient location on the saliency map. Given the input color image, different feature maps are produced by applying linear filters to a specific stimulus property like color, orientation, or intensity. The feature maps are then combined to give three Conspicuity Maps and finally, a saliency map is computed for the Conspicuity maps (Walther and Koch, 2006). A winner-take-all (WTA) mechanism finds the coordinates of the most salient location after scanning the saliency map. Inhibition of return (IOR) of a circular shape with fixed radius is applied around the attended location in the saliency map. Subsequent iteration of the WTA network attends to the locations in decreasing order of saliency. The model is verified in several human psychophysical experiments (Itti, 2005; Peters et al., 2005).

##### Saliency-Based Bottom-up Attention Model

The input image *I* is first sub-sampled into a Gaussian pyramid. The Gaussian pyramid is created by convolution of input image *I* with a set of Gaussian filters and subsampling with a decimation factor of 2 to generate a sequence of reduced resolution images. This process is repeated and a total of 9 different scales are created σ = [0, .., 8] (level: 0 corresponds to the original input image; Walther and Koch, 2006). At level σ, the resolution of the image is 1/2^σ^ of the original image. For level eight i.e., σ = 8, the resolution equals to 1/256th of the input image I and (1/256)^2^ of the total no of pixels of the input image.

The intensity map *M*_*I*_ is computed by adding the r (red), g (green), b (blue) values of the color image (Walther and Koch, 2006).

(1) $$\begin{array}{l} M_{I}=\left(r+g+b\right)\;/3 \end{array}$$

Intensity Pyramid *M*_*I*_(σ) is created by repeating the same operation at different levels.

Using the Image Pyramid, blue-yellow (BY), and red-green (RG) opponency maps are created at every level (Walther and Koch, 2006).

(2) $$\begin{array}{l} M_{RG}=\frac{r-g}{\text{max}\left(r,g,b\right)} \end{array}$$

(3) $$\begin{array}{l} M_{BY}=\frac{b\;-\;\text{min}\left(r,g\right)}{\text{max}\left(r,g,b\right)} \end{array}$$

Orientation maps *M*_θ_ are obtained from intensity maps by convolving the various levels of Intensity pyramids with Gabor filters (Walther and Koch, 2006):

(4) $$\begin{array}{l} M_{\theta }\left(\sigma \right)=||M_{I}\left(\sigma \right)*G_{0}\left(\theta \right)||+||M_{I}\left(\sigma \right)*G_{\pi /2}\left(\theta \right)||, \end{array}$$

Multiscale feature extraction is done by across scale subtraction Θ between two maps levels c and s in these pyramids. Across scale subtraction Θ, is defined as interpolation to the finer scale, followed by point-to-point subtraction between maps. In other words, it is the difference between fine and coarse scale features of an image. Using many different values for c and s provides truly multiscale feature extraction (Walther and Koch, 2006).

(5) $$F_{l,c,s}=\;Ɲ\left(\left|M_{l}\left(c\right)\Theta M_{l}\left(s\right)\right|\right)\forall l\in L=L_{I}\cup L_{C}\cup L_{O}$$

where

$$\begin{array}{l} L_{I}=\left\{I\right\},L_{C}=\left\{RG,BY\right\},\;L_{O}=\left\{0^{o},\;45^{0},90^{0},135^{0}\right\} \end{array}$$

Ɲ (·) is a non-linear iterative operator, which promotes local completion among neighborhood salient locations. At each iteration step, self-excitation and neighbor-induced inhibition is implemented with a “difference of Gaussians” filter and then followed by rectification.

Using across scale addition ⊕ features maps are then summed over then normalized again.

(6) $$\begin{array}{l} \bar{F_{l}}=Ɲ\left({⊕}_{c=2}^{4}{⊕}_{s=c+3}^{c+4}F_{l,c,s}\right)\forall l\in L \end{array}$$

Three conspicuity maps of general features are created: one for intensity, one for color and one for orientation (Walther and Koch, 2006).

(7) $$\begin{array}{l} C_{I}=\bar{F_{I}}, \end{array}$$

(8) $$\begin{array}{l} C_{C}=Ɲ\left(\sum_{l\in L_{c}}\bar{F_{l}}\right), \end{array}$$

(9) $$\begin{array}{l} C_{o}=Ɲ\left(\sum_{l\in L_{o}}\bar{F_{l}}\right). \end{array}$$

Then, a single saliency map is created by combining all the three conspicuity maps (Walther and Koch, 2006).

(10) $$\begin{array}{l} S=\;\frac{1}{3}\sum_{k\in \left\{I,C,O\right\}}C_{k} \end{array}$$

Within the saliency map, different locations compete for saliency. The most salient location is selected for attention. Inhibition of Return (IOR) is applied to the selected area for some time within a given radius. In the second iteration, the remaining locations compete for saliency and the second most salient location is selected. Thus, a saccadic scan path is created on the image in order of decreasing saliency (Walther and Koch, 2006).

**Figure 2:**
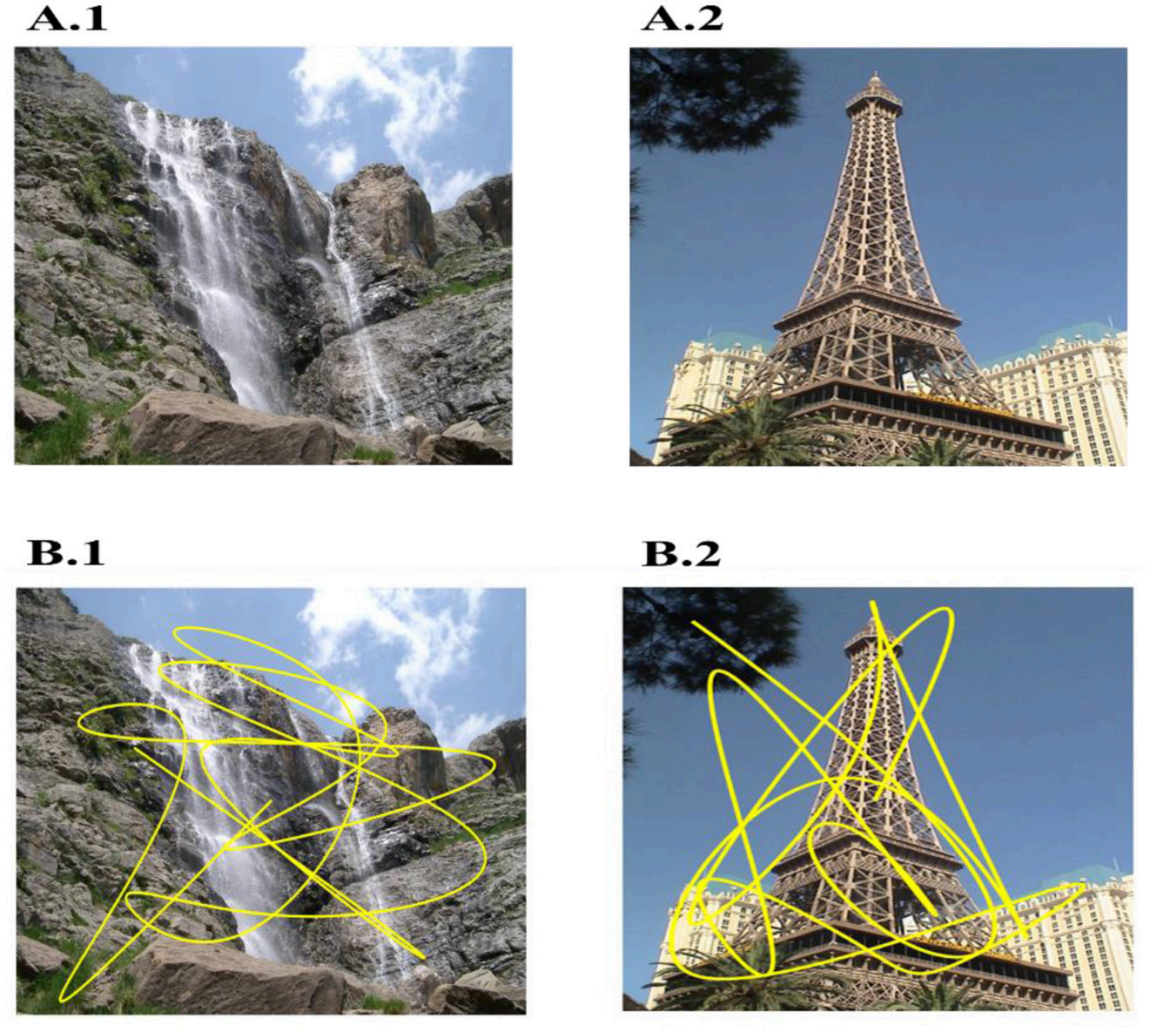
Two sample images used **(A1,A2)** and overlapped trajectory (Yellow) generated on them **(B1–B2)** by bottom-up model of attention. Sample images are taken from: Caltech-256 Object Category Dataset. (No copyright permission is required).

In the simulation, to match with the experimental paradigm, we used 36 novel images wherein each image is presented twice, for 10 s each to produce the saccade trajectory (Killian et al., 2012). Two sample figures with trajectories superimposed on them are shown in Figures 2A,B. Image Source: Caltech-256 Object Category Dataset (Griffin et al., 2007).

#### Saccade Direction Layer (SD) Layer

Saccade trajectory generated from the SG stage is passed to the saccade direction layer (SD). SD layer encodes the animal's current saccade direction as given in Equation (11). The response of i^th^ cell of SD layer is computed as the animal's current saccade direction projection on the i^th^ preferred direction given as.

(11) $$\begin{array}{l} \alpha_{i}=\text{cos}\left(\theta -\theta_{i}\right) \end{array}$$

θ, θ_*i*_ are the current direction and the preferred direction of i^th^ SD cell, respectively.

#### Path Integration (PI) Layer

SD layer connects to PI layer via one-to-one connections. The response of the i^th^ PI cell is given as,

(12) $$\eta_{i}=\text{A }*\text{ sin}[\int 2\pi (f_{o}+\beta s\alpha_{i})dt]$$

β is a spatial scaling parameter, A = Amplitude of oscillations. s is the speed of the Saccade. *f*_*o*_ is the base frequency of the PI neuron. The i^th^ PI neuron is then thresholded by the following equation.

(13) $$\begin{array}{l} \eta_{i}^{Thr}=H\left(\eta_{i}-\varepsilon_{\eta }\right).\eta_{i} \end{array}$$

where, H is Heaviside function and ε_η_the threshold value.

Power of oscillation is given in decibel as:

(14) $$\begin{array}{l} P=20*log_{10}\left(A\right) \end{array}$$

#### Output Layer (SC)

PI values project to SC layer via the weight stage (W–^PC^). Weights (W^PC^) from PI layer to SC layer are computed by performing Principal Component Analysis (PCA) over η ^Thr^. PCA was done by extracting the top few eigenvectors of the covariance matrix of the $\eta_{i}^{Thr}$. The response of the i^th^ neuron in the SC layer is computed as:

(15) $$\begin{array}{l} O_{i}=\;\overset{N}{\underset{j=1}{\sum }}H\left[\left(W_{ij}^{PC}.\eta_{j}^{Thr}\right)-\varepsilon_{SC}\right] \end{array}$$

where, H is Heaviside function.

N is the number of PI neurons, ε_*SC*_ is the threshold value.

**Figure 3:**
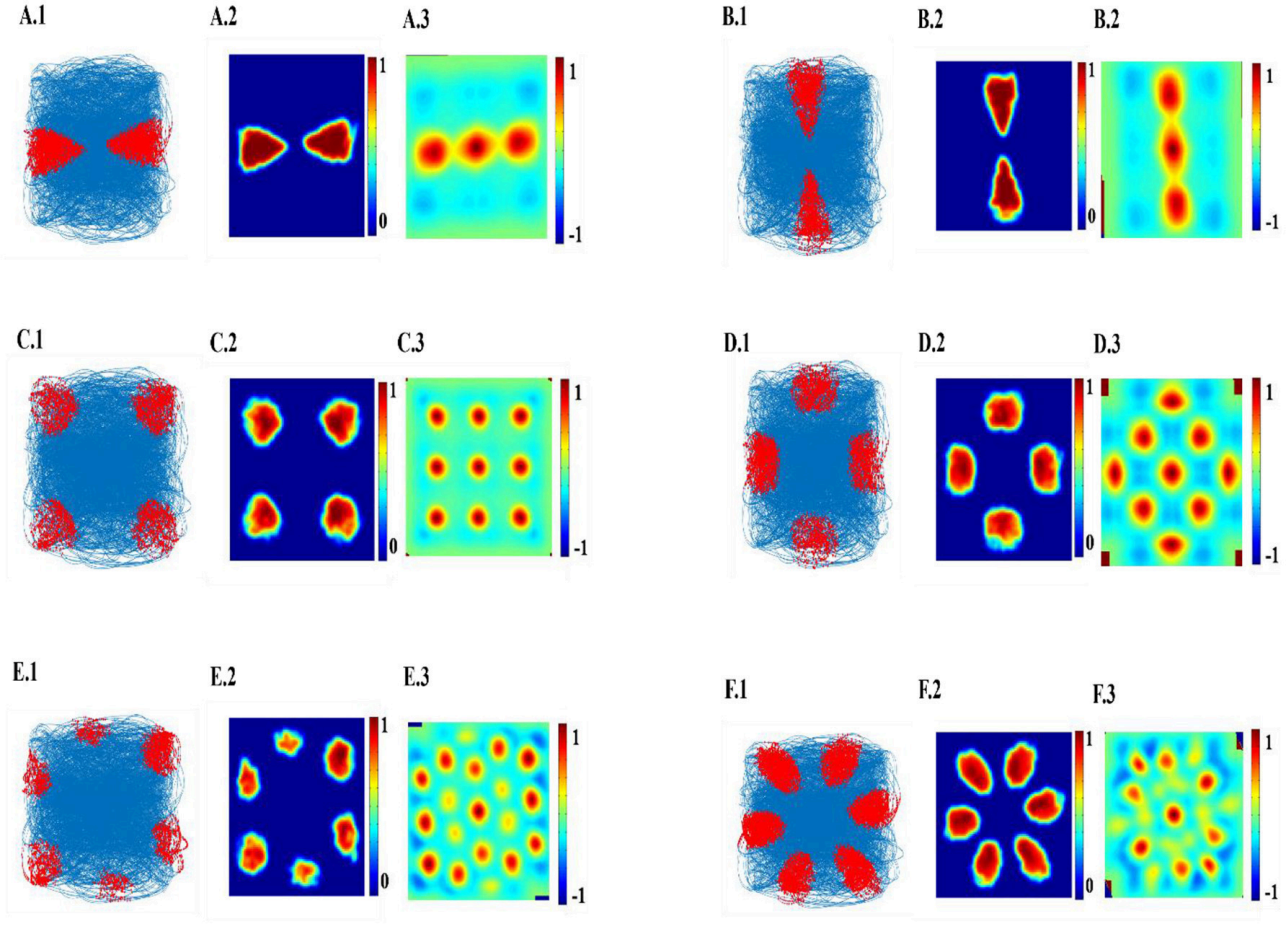
Spatial representations from SC layer: **(A–F)** Firing fields (Left): Blue is the trajectory of the Saccade and red dots are the spike locations; Firing Rate map (Middle): red is peak rate and blue is no firing; Autocorrelation map (Right) of SC layer neuron in SVDON-PCA model.

The top few components of the computed principal component (PC) will be shown to reveal a variety of spatial cell-like responses including grid cells (Figure 3). Spatially periodic firing emerges due to the inherent periodicity in the PC weights. Hexagonal grid-like activity is shown by the neurons whose peaks are separated by ≈ 60° (PC = 6).

### SVDON-NPCA Architecture

SVDON-NPCA model has a similar architecture to SVDON-DPCA. Here a neural network implementation of PCA is used instead of direct PCA, we replaced 1D SD layer with a Self-Organizing Map (SOM) where a two-dimensional layer of neuron is used to represent saccade direction. Lateral Anti-Hebbian Network (LAHN) is the network implementation of PCA (Foldiak, 1989) which is used to extract the optimal features from the input data by variance maximization principle. The changes made in this model permit us the leverage to study grid cells from network perspective.

Below we explain the SOM architecture of SD layer and the LAHN layer in detail.

#### Saccade Direction Layer

Like in SVDON-DPCA, here also saccade velocity vectors are passed on to the SOM in the next layer to obtain a direction map. SOM neuron response is given as:

(16) $$\begin{array}{l} \theta_{\text{SD}}=\psi^{T}W \end{array}$$

ψ^*T*^ = [sin(θ), cos(θ)] where θ is current direction of navigation, is given as input given to SOM.

W = Normalized afferent weight matrix of SOM.

#### Lateral Anti Hebbian Network (LAHN) Layer

LAHN is an afferent Hebbian and lateral anti-Hebbian unsupervised neural network, which extracts the variance feature from the input.

The network is described as

(17) $$\begin{array}{l} \xi_{i}\left(t\right)=\sum_{j=1}^{m}q_{ij}\chi_{j\;}\left(t\right)+\sum_{k=1}^{n}w_{ik}\xi_{k}\left(t-1\right) \end{array}$$

q is the weight of the afferent connection

χ is input PI value

m is the input dimension

n is the number of LAHN neurons, ξ is the network response

Hebbian rule is used to update afferent connection and a anti-Hebbian rule is used to update lateral connection as described below

(18) $$\begin{array}{l} \Delta w_{ik=-}\eta_{L}\xi_{i}\left(t\right)\xi_{k}\left(t-1\right) \end{array}$$

(19) $$\begin{array}{l} \Delta q_{ij}=\eta_{F}\left[\chi_{j\;}\left(t\right)\xi_{i}\left(t\right)-q_{ij}{\xi_{i}}^{2}\left(t\right)\right] \end{array}$$

Where η_*L*_ *and η*_*F*_ lateral and forward learning rate, respectively. After training network weights of LAHN network converges to subspace of principal components of input vector.

### Gridness Measure

The hexagonal gridness measure is quantified using Hexagonal Gridness score (HSG) on firing fields of each neuron. HGS is computed using Equations 20, 21 (Hafting et al., 2005).

(20) $$r(\tau_{x},\tau_{y})=\frac{M\sum_{x,y}\lambda (x,y)\lambda (x-\tau_{x},y-\tau_{y})-\sum_{x,y}\lambda (x,y)\sum_{x,y}\lambda (x-\tau_{x},y-\tau_{y})}{\sqrt{\{M\sum_{x,y}\lambda {\left(x,y\right)}^{2}-[\sum_{x,y}\lambda (x,y){]}^{2}]\}\{M\sum_{x,y}\lambda {\left(x-\tau_{x},y-\tau_{y}\right)}^{2}-{\left[\lambda (x-\tau_{x},y-\tau_{y})\right]}^{2}\}}}$$

r is an autocorrelation map, λ (x, y) is firing rate at (x,y) location of the rate map, M is the total no of pixels in the rate map, τ_*x*_ and τ_*y*_ correspond to x and y coordinates with a spatial lag

(21) $$\begin{array}{l} HGS=\;\text{min}[\text{cor}(\text{r},{\text{r}}^{60^{0}}),\text{cor}(\text{r},{\text{r}}^{{\text{120}}^{\text{0}}})]-\text{max}[\text{cor}(\text{r},{\text{r}}^{{\text{30}}^{\text{0}}}),\\ \text{ cor}(\text{r},{\text{r}}^{{\text{90}}^{\text{0}}}),\text{cor}(\text{r},{\text{r}}^{{\text{150}}^{\text{0}}})] \end{array}$$

HGS stands for Hexagonal Gridness Score;

r° is the autocorrelation map rotated by θ degree;

*cor*(·) stands for correlation function;

*min*(·) function returns the minimum of its two arguments.

## Results

### SVDON-DPCA

SC neuron activity is mapped onto saccadic trajectory. Figure 3 shows the firing field, firing rate map and autocorrelation map of the six SC layer neuron receiving the first six principle components.

**Figure 4:**
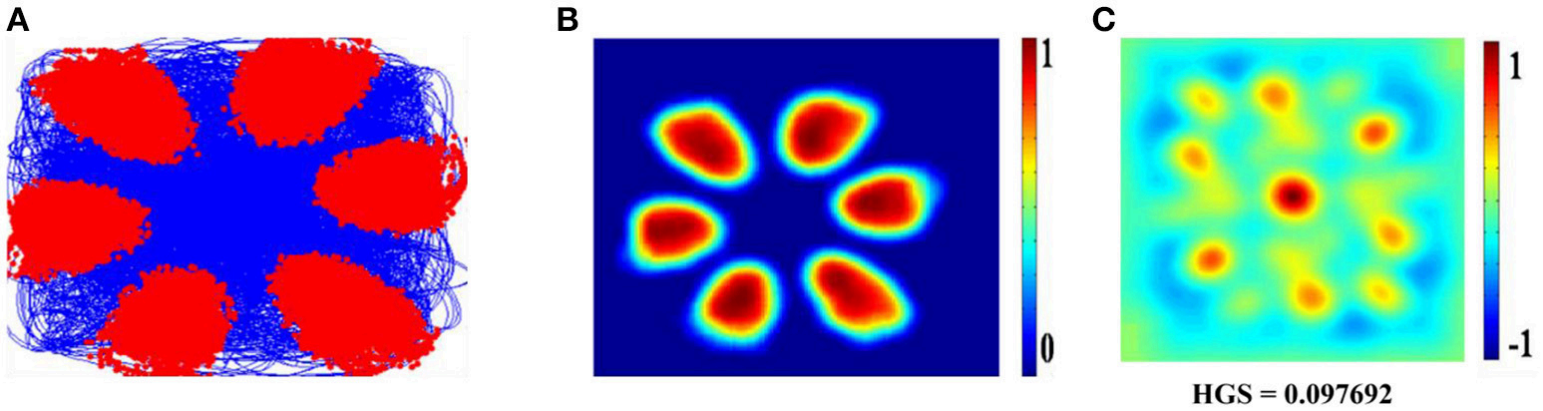
Spatial representations from sixth SC layer neuron: Firing field **(A)**: Blue is the trajectory of the Saccade and red dots are the firing locations; Firing map **(B)**: red is peak rate and blue is no firing; Autocorrelation map **(C)** of the sixth SC layer neuron in SVDON- PCA model.

Hexagonal Firing field is shown by the neuron which received the sixth principal component (Figure 4). A neuron is considered to be canonical if it had a HGS>0 (Hafting et al., 2005).

### Oscillation Power and β Modulation

**Figure 5:**
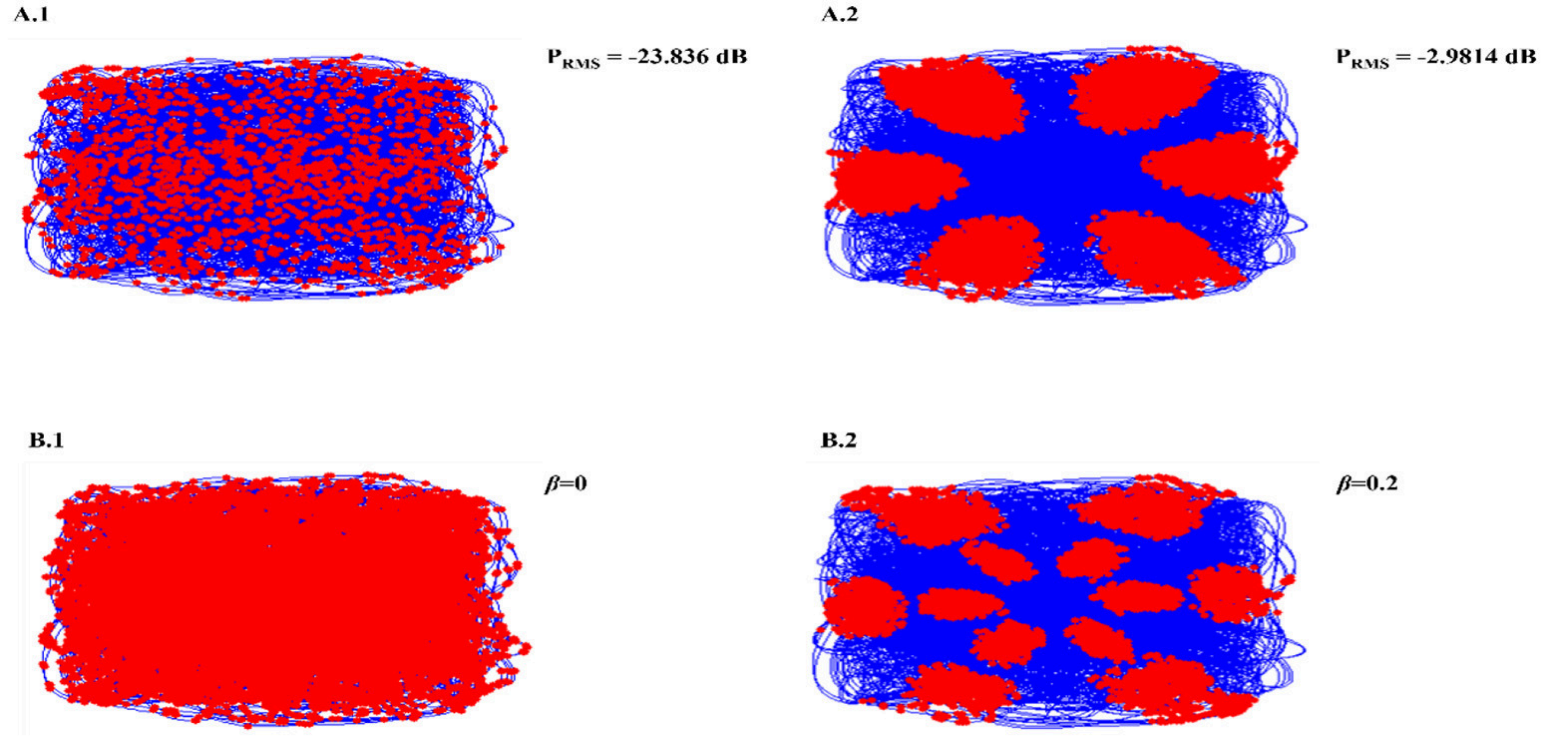
Oscillation power modulation **(A)**: loss of grid field formation on reducing oscillation power **(A1)**; grid field reemerged as the oscillation power was restored **(A2)**. β modulation **(B)**: loss of grid field when β = 0 **(B1)**, grid field reemerged when β = 0.2.

Necessity of oscillations to produce grid patterns was contested by varying the oscillatory power in the PI layer. Power is a function of amplitude of oscillations (Equation 14). Hence by changing the amplitude variable (Equation 12), we were able to change the power of the oscillations. By reducing oscillation power there was a loss of Grid Field formation, but grid field reemerged as the oscillation power was restored (Figure 5A).

We also analyzed the criticality of the modulation of oscillations in the PI layer by varying the β parameter (Equation 12). Similar loss of grid field is seen with β modulation (Figure 5B).

### SVDON-NPCA

**Figure 6:**
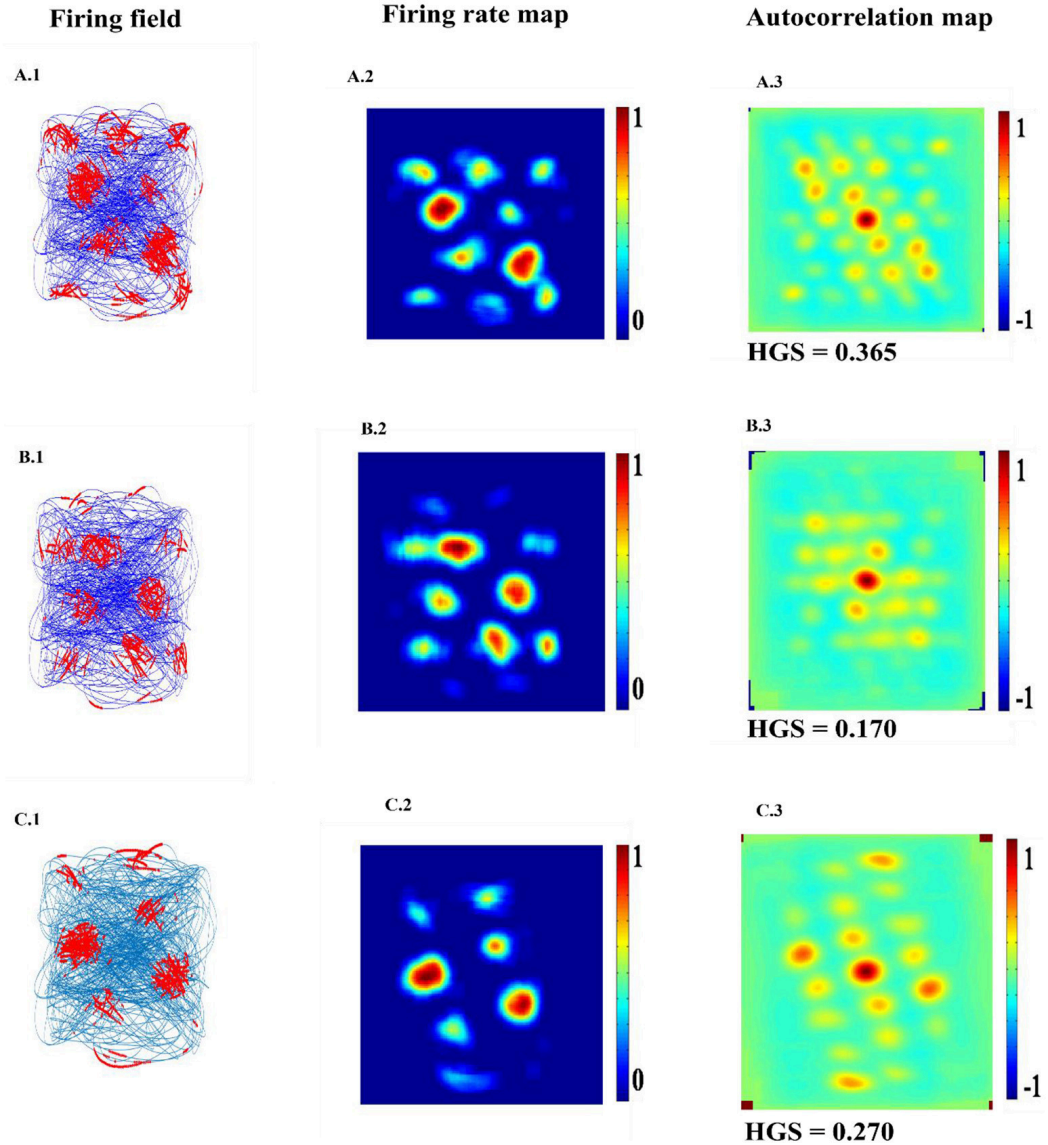
Spatial representations of three different neurons **(A–C)** from LAHN (SC) layer. Left Column is firing field of the neurons **(A1–C1)**; Middle Column is the firing rate of the neurons **(A2–C2)**; and the Right column is autocorrelation map of the neurons **(A3–C3)**.

LAHN (SC) network of the model shows the spatially periodic firing in Figure 6. The firing fields of LAHN (SC) neurons have more heterogeneity compared to PCA.

### Spatial Characteristics of Grid Cells

**Figure 7:**
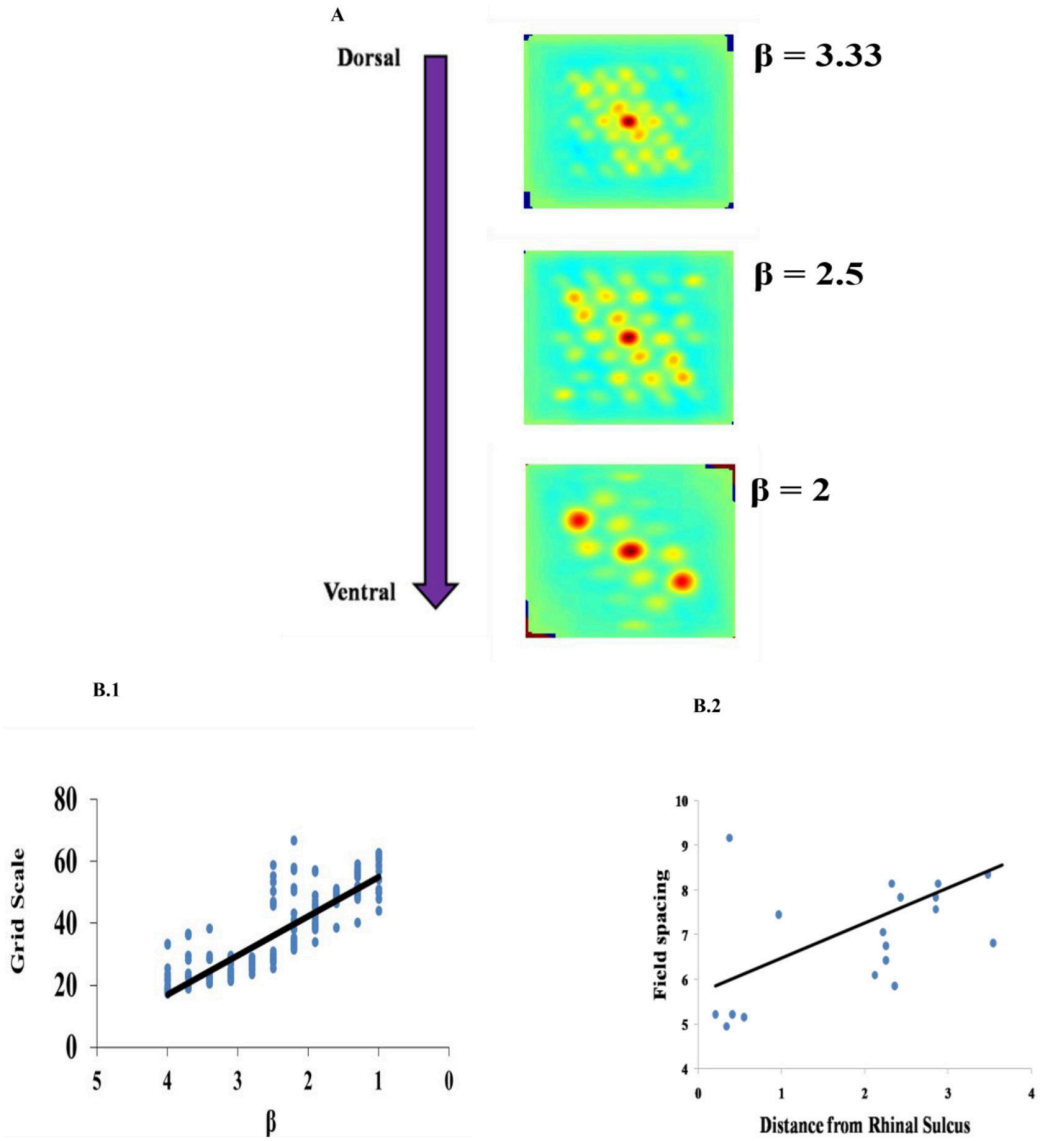
Effect of modulation factor β. **(A)** Grid Scale gradient is captured by varying spatial scale parameter β. **(B)** Comparison between the model and the experiment. **(B1)** Grid scale variation in the model by varying β (Grid scale is averaged for 10 trajectories). **(B2)** Empirically observed grid scale variation at different locations of medial to dorsal rhinal sulcus axis (Killian et al., 2012).

Grid Scale variation across the dorso-ventral axis of MEC has been demonstrated in experimental studies of rodent navigation (Brun et al., 2008; Stensola et al., 2012). A similar gradient was observed in the case of saccadic trajectories also (Killian et al., 2012). To capture this in the model, we varied the β parameter as shown in Figure 7A. This variation is shown in Figure 7B1 contrasted with the experiment results in Figure 7B2. Grid scale was quantified by computing the distances between the six inner hexagonal vertices from the central peak in the autocorrelation map, minimum of these values represents the grid scale. (Burn, Solstad et al., 2008).

### Predicting Place Cells Activity

**Figure 8:**
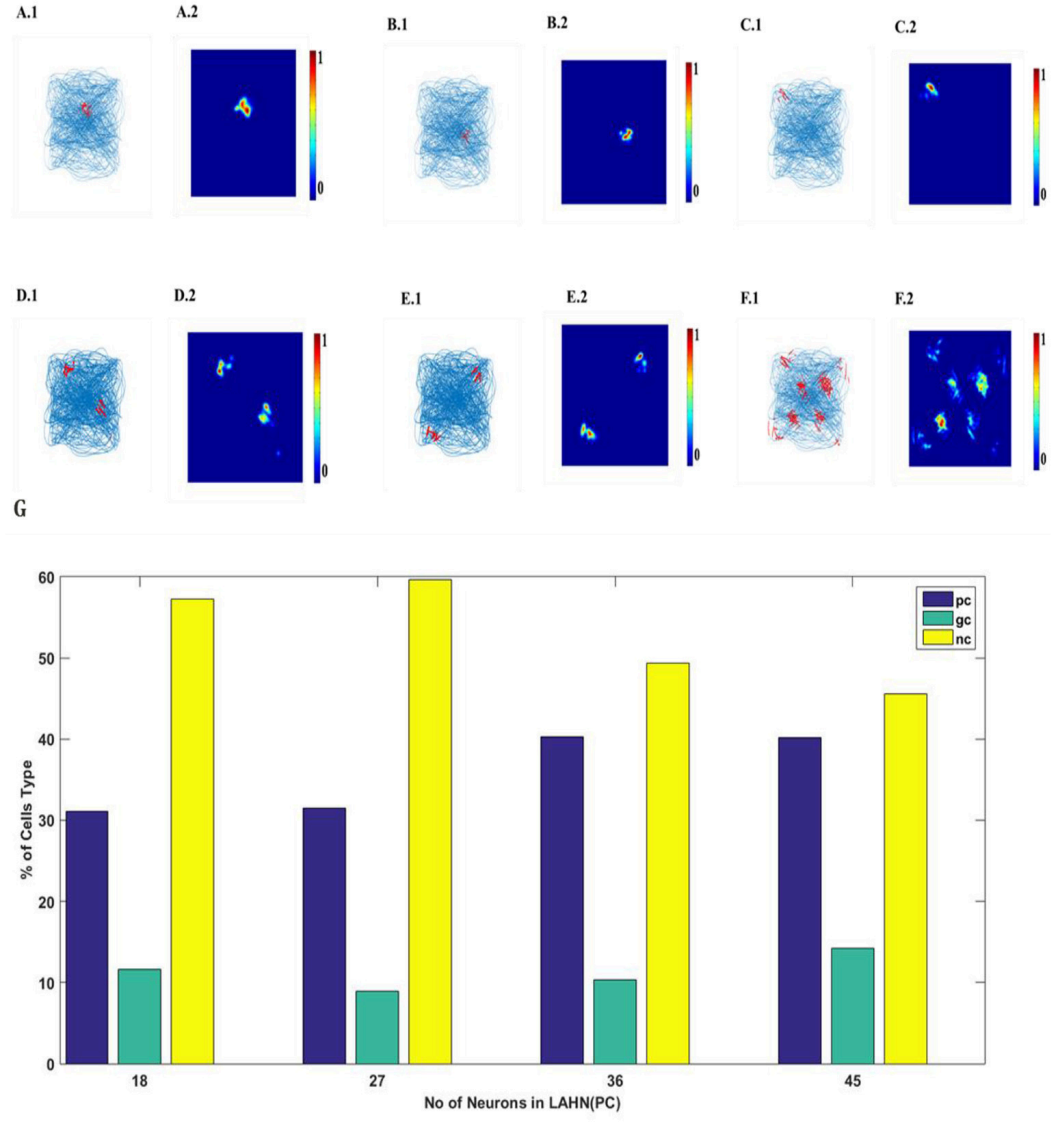
LAHN (PC) layer activity of six different neurons. **(A1–F1)** firing field (blue is the trajectory of the Saccade and red dots are the spike locations) and **(A2–F2)** firing maps (red is peak rate and blue is no firing) of 6 different neurons in LAHN(PC). Characterization of LAHN (PC) layer showing the % of cells type vs. the number of neurons in layer. Here PC is Place cells, GC is Grid Cells and NC is non-spatial cells.

SVDON-NPCA model is capable of exhibiting place cell like activity on saccadic space when a second LAHN (place cells) layer is added after the first LAHN (SC Layer in Figure 1). Experimental Studies have shown that the number of neurons (Akdogan et al., 2011) in rat CA1 region is about 90 percent of EC (considering only layer 2 and layer 3 of MEC as they form major afferent synapse with CA1). Accordingly, a similar ratio of neurons is kept in LAHN (SC) and LAHN (PC) layers. The output the LAHN (SC) is passed on to LAHN(PC) layer and the activity of the LAHN(PC) layer is observed, LAHN(PC) neurons showed a highly localized firing activity similar to that of place cells. To qualify a neuron as a place cell, the number of peaks in autocorrelation map is examined. A cell is characterized as a place cells if the number of peaks in autocorrelation is one (Soman et al., 2018b) due to its localized firing field and lack of spatial periodicity. LAHN (PC) layer also predicted spatial cells that showed spatial periodicity as shown in Figure 8.

### Place Activity on Single Image

**Figure 9:**
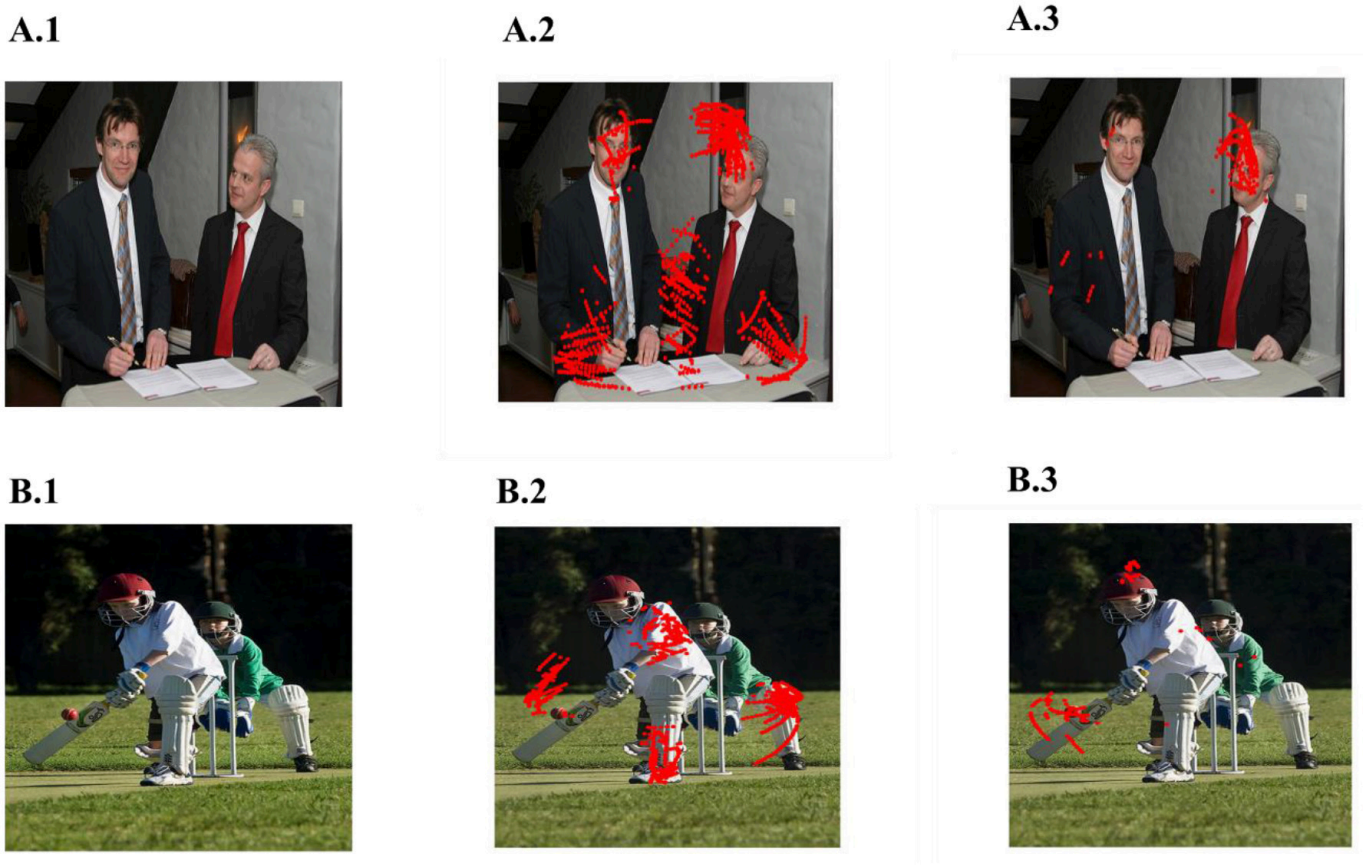
Spatial cell response to a single image. **(A1,B1)** Images given as the input. **(A2,B2)** are the outputs of LAHN(SC) and **(A3,B3)** are the outputs of LAHN(PC). Red dots are the firing locations on the image (Images source is ImageNet: A Large-Scale Hierarchical Image Database).

In the simulations described in the previous sections, the trajectories were obtained from a large number of images and grid and place cell responses are generated from that combined trajectory. In this section, we generate a trajectory from single images and superimpose the place cells generated from that trajectory back on the original image. The objective is to see if the grid and place cells obtained from the trajectory correspond to salient features/objects in the image. To produce saccade trajectory, we presented a single image for 125 s to the saccade trajectory generating model. The trajectory is then used to train the model of Figure 1 with the added module of LAHN (PC). Figure 9 shows results from two images. Place cells obtained from these images are indeed localized on salient objects in the image such as face of the person (Figure 9A3) or the bat of the batsman (Figure 9B3). Image source : ImageNet: A large scale hierarchical Image Database (Deng et al., 2009).

## Discussion

We present two models: SVDON-DPCA and SVDON-NDPCA to capture the saccadic representation based on the input saccadic trajectory formed on a series of images. In the SVDON-DPCA, we have shown the formation of hexagonal grid cell periodicity using Direct PCA. The model is simple and transparent and gives an insight into the origins of the grid cell spatial periodicity. In SVDON—NPCA, we used LAHN layer instead of direct PCA to produce hexagonal grid cells. This substitution is made since LAHN is based on a biologically more plausible learning mechanisms viz. lateral anti-Hebbian and afferent Hebbian learning, than the PCA. LAHN weight vectors have been shown to converge to the principal component subspace (Foldiak, 1989). Such a connectivity pattern is critical for a self-organization process because the excitatory Hebbian connections to a neuron could essentially correlate its activity to the input features and the lateral inhibitory connections could ensure competition among the ensemble of neurons to extract out diverse features of the input. This sort of connectivity pattern is biologically plausible and is consistent with the empirically reported GABArgic interneuron connections between the stellate cells in the superficial layer of the medial entorhinal cortex (Couey et al., 2013). In addition to this, anti-Hebbian network has been previously shown to encode the input data optimally by minimizing a representation error/multidimensional scaling cost function (Pehlevan et al., 2015). Hence the model gives insight to the self-organization among the grid/quasi-grid units and the relevance of such a connectivity pattern for optimal spatial representation.

The primate visual system scanning a complex visual scene seems to employ a serial search strategy. In primates, object identification and spatial analysis of the image is achieved by a series of rapid saccadic eye movements. Saccades occur reflexively whenever the eyes are open and also can be elicited voluntarily (Liversedge and Findlay, 2000). Different visual locations compete for activity and the strongest response draws the visual attention. These are called visually salient locations (Slllito et al., 1995; Sillito and Jones, 1996; Levitt and Lund, 1997). The bottom-up model used in our architecture for saccade generation is based on a similar approach that generates a two-dimensional saliency map of the visual environment. Experimental evidence has shown the existence of neural maps in the pulvinar, the superior colliculus, and the intraparietal sulcus which encode for the saliency for visual stimulus (Robinson and Petersen, 1992; Gottlieb et al., 1998; Colby and Goldberg, 1999; Rockland et al., 1999). The results from the models discussed above are similar to the grid cells that have been reported in the rat and bat during locomotion (Hafting et al., 2005; Yartsev et al., 2011). These results imply that ideas of spatial representation for navigation also apply to complex visual scene analysis because these results show that visual exploration of space can give rise to representations for that space even without performing active navigation over the corresponding physical space.

The results produced by our model are consistent with the experimental literature. The variation in the gradient of the grid scale along the dorso-ventral axis of the entorhinal cortex is reported in the experimental literature (Brun et al., 2008; Stensola et al., 2012). It is shown that the grid scale varied from low to high value with the distance from the rhinal sulcus (Killian et al., 2012), which is consistent with a dorsal-ventral gradient in rodents and bats for navigation (Hafting et al., 2005; Yartsev et al., 2011). To incorporate this in our model, we varied the parameter β in Path Integration layer. β determines the modulation factor for the path integration neuron. Even though the model captures gradient in the grid scale by varying the β parameter, it does not explain the modular formation of grid cells along the dorso-ventral axis of MEC where, in each module, grid cells with the same grid scale and grid orientation and different grid phases occur and the grid scale varies across the modules in a geometric progression fashion with a scale ratio of √2. Here, the grid scale can be fitted to any ratio by varying the β parameter accordingly.

From the model results it is understood that oscillations are critical for the grid cell generation. Oscillations introduce the first spatial periodicity by encoding the position information in their respective phase. This periodicity is further transformed to grid-like representations in the higher layer. It was empirically shown in rats that abolition of theta activity in the MEC causes the grid representations to fade out (Giocomo et al., 2007). In the model, we tested the same by decreasing the oscillatory power of the path integration neurons and found a corresponding disruption in the grid representations (Figure 5A). However, we would like to pose this oscillation and grid cell phenomenon as a prediction from the model since this phenomenon has not been reported yet in saccade studies. Further analysis also showed the criticality of modulation in oscillation for the grid formation. Modulation is set to off by making β set to zero. No grid fields are observed in that condition. When β is set to a non-zero value, grid fields start to appear (Figure 5B).

Place cell like activity is predicted by the model on the scaddic space upon adding an extra layer of LAHN(PC) on top of LAHN(SC). Although place cell like activity have not been experimentally reported yet. On giving saccadic trajectory of single images as a input to the model, LAHN(PC) neurons fired on naturally significant locations on image like the face and the bat of the batsman shown in Figure 9. These predictions are consistent with the recent observation that navigation in physical space can be just one of the many roles played by place cells, grid cells and other hippocampal spatial cells.

Taken together, these models computationally try to explain the generation of grid cell representations in the entorhinal cortex based on the saccade trajectory generated during visual exploration of a natural scene. They also predicts the place cells like activity on saccadic space. The grid field generated by the SVDON—DPCA does not have the central firing field which we consider as the limitation of this model, this limitation is overcome by the second model viz. SVDON—NPCA. In the future work, we would like to extend this model by including visual and locomotor input along with the saccadic input and search for the possible existence of joint representations arising out of the spatial navigation of the physical space and saccadic exploration of the image space. Virtual Reality (VR) environments offer a convenient setting for conducting such simulation studies.

## Author Contributions

AC contributed to performing simulations and analysis. KS contributed to simulation design and manuscript preparation. VSC contributed to simulation design and manuscript preparation.

### Conflict of Interest Statement

The authors declare that the research was conducted in the absence of any commercial or financial relationships that could be construed as a potential conflict of interest.
